# Unleashing the Power of Biosensors and Artificial Intelligence in Dermatology

**DOI:** 10.1093/asjof/ojae030

**Published:** 2024-04-24

**Authors:** Diala Haykal

In an era marked by exponential technological advancements, the convergence of biosensors and artificial intelligence (AI) presents a transformative opportunity to revolutionize the landscape of dermatological care.

In today’s world, biosensors play a prevalent role in biomedical diagnostics and extend their utility across diverse sectors in our clinical practice, and biomedical research. Biosensors provide exceptional insights into skin health and disease by allowing early identification of skin conditions through the detection of specific biomarkers, which helps in prompt interventions. The sensors offer immediate monitoring of disease advancement and treatment effectiveness, enabling tailored treatment modifications and enhanced patient care.^1^ Dermatologists can use these sensors to gather real-time information on different biomarkers, enabling early detection and treatment of conditions, such as skin cancer and inflammatory disorders. Additionally, the noninvasive quality of biosensors increases patient comfort and adherence, ultimately enhancing healthcare results. Biosensors offer objective data on skin health parameters, aiding in personalized medicine and improving treatment strategies.^2^

Moreover, the incorporation of AI enhances the diagnostic and prognostic abilities of dermatologists. AI algorithms, trained on extensive datasets, can quickly analyze intricate patterns and images, assisting in the precise diagnosis of skin lesions and diseases. AI-driven decision support systems can help clinicians create personalized treatment plans based on individual patient profiles, improving treatment effectiveness and reducing side effects.^3^

The combination of biosensors and AI improves clinical practice and enables patients to take an active role in their healthcare. Wearable biosensor devices with AI applications allow individuals to track their skin health in real time, facilitating early intervention and preventive actions. This proactive strategy not only lessens the strain on healthcare systems but also promotes a culture of well-being and self-care among the population.

Dermatology biosensors have different levels of sensitivity, with some able to detect very low levels of biomarkers, making them useful for early disease detection. The long-term stability of sensors is critical for their reliability in clinical settings, where environmental factors and sample conditions can significantly affect their performance. Biosensors may be limited in terms of specificity, cross-reactivity with other compounds, and potential interference from biological matrices.^4,5^ To overcome these challenges, continuous research and development are needed to enhance sensor design, improve selectivity, and optimize performance characteristics. Despite their limitations, biosensors nevertheless hold great potential for improving dermatological diagnostics and personalized patient care. Specifically, tattoo sensors, by leveraging their low cost, highly biocompatible injectable technology, address these challenges effectively. Their design not only ensures resilience against varying conditions but also democratizes health monitoring using smartphones for data analysis. The convergence of stability and accessibility facilitates the implementation of continuous, multiplexed health monitoring, which efficiently and practically introduces advanced healthcare functionalities to lower income regions.^6,7^

Given these advancements, healthcare stakeholders must prioritize adopting and investing in the incorporation of biosensors and AI-based tools into dermatological practice. Utilizing technology can lead to a new phase of precise dermatology marked by early detection, tailored treatment, and enhanced patient results.^8^ Overall, biosensors and AI-based tools have the potential to revolutionize dermatological practice by providing clinicians with valuable insights into skin health and disease processes, ultimately leading to improved patient outcomes and quality of life.

## References

[1] Bhalla N, Jolly P, Formisano N, Estrela P. Introduction to biosensors. Essays Biochem. 2016;60(1):1–8. doi: 10.1042/EBC20150001 27365030 PMC4986445

[2] Zhang L, Guo W, Lu Y. Advances in cell-free biosensors: principle, mechanism, and applications. Biotechnol J. 2020;15(9):2000187. doi: 10.1002/biot.202000187 32667120

[3] Groh M, Badri O, Daneshjou R, et al Deep learning-aided decision support for diagnosis of skin disease across skin tones. Nat Med. 2024;30(2):573–583. doi: 10.1038/s41591-023-02728-3 38317019 PMC10878981

[4] Wang Y, Zhou W, Shen C, Jiang G, Yang C. Flexible and printable integrated biosensors for monitoring sweat and skin condition. Anal Biochem. 2023;661:114985. doi: 10.1016/j.ab.2022.114985 36414087

[5] Fang W, Wu J, Cheng M, et al Diagnosis of invasive fungal infections: challenges and recent developments. J Biomed Sci. 2023;30(1):42. doi: 10.1186/s12929-023-00926-2 37337179 PMC10278348

[6] Dhond K, Hu Y, Yetisen AK. Dermal tattoo biosensors. Dermatol Heidelb Ger. 2023;74(10):819–821. doi: 10.1007/s00105-023-05195-6 PMC10516771 37450053

[7] Yetisen AK, Moreddu R, Seifi S, et al Dermal tattoo biosensors for colorimetric metabolite detection. Angew Chem Int Ed Engl. 2019;58(31):10506–10513. doi: 10.1002/anie.201904416 31157485

[8] Mazur F, Tjandra AD, Zhou Y, Gao Y, Chandrawati R. Paper-based sensors for bacteria detection. Nat Rev Bioeng. 2023;1(3):180–192. doi: 10.1038/s44222-023-00024-w 36937095 PMC9926459

